# Diet induced thermogenesis

**DOI:** 10.1186/1743-7075-1-5

**Published:** 2004-08-18

**Authors:** Klaas R Westerterp

**Affiliations:** 1 Department of Human Biology, Maastricht University, PO Box 616, 6200 MD Maastricht, The Netherlands

**Keywords:** carbohydrate, protein, fat, alcohol, satiety

## Abstract

**Objective:**

Daily energy expenditure consists of three components: basal metabolic rate, diet-induced thermogenesis and the energy cost of physical activity. Here, data on diet-induced thermogenesis are reviewed in relation to measuring conditions and characteristics of the diet.

**Methods:**

Measuring conditions include nutritional status of the subject, physical activity and duration of the observation. Diet characteristics are energy content and macronutrient composition.

**Results:**

Most studies measure diet-induced thermogenesis as the increase in energy expenditure above basal metabolic rate. Generally, the hierarchy in macronutrient oxidation in the postprandial state is reflected similarly in diet-induced thermogenesis, with the sequence alcohol, protein, carbohydrate, and fat. A mixed diet consumed at energy balance results in a diet induced energy expenditure of 5 to 15 % of daily energy expenditure. Values are higher at a relatively high protein and alcohol consumption and lower at a high fat consumption. Protein induced thermogenesis has an important effect on satiety.

In conclusion, the main determinants of diet-induced thermogenesis are the energy content and the protein- and alcohol fraction of the diet. Protein plays a key role in body weight regulation through satiety related to diet-induced thermogenesis.

## Introduction

Diet induced thermogenesis (DIT) can be defined as the increase in energy expenditure above basal fasting level divided by the energy content of the food ingested and is commonly expressed as a percentage. It is, with basal metabolic rate and activity induced thermogenesis, one of the three components of daily energy expenditure. Although DIT is the smallest component, it could play a role in the development and/or maintenance of obesity. De Jonge and Bray [1] evaluated 49 studies that compared DIT in subjects who were obese with those who were lean. Of 29 studies, in which the subjects with obesity had a significantly higher body mass index compared with the lean individuals, and the two groups were well matched for age, 22 studies reported a significantly lower DIT in the subjects with obesity. Granata and Brandon [2] suggested the theory that DIT is reduced in obesity appears to be attractive and plausible, yet discrepant findings persist in the literature and research has uncovered numerous flaws and concerns regarding the methods used to measure and calculate DIT.

Methodological issues include: was the baseline appropriate, what was the energy content and nutrient composition of the test food consumed, what was the duration of the postprandial measurement period, and what was the error associated with the calculation of DIT from the measured energy expenditure. Weststrate et al [3] investigated whether repeated measurements varied with time of day and found no significant diurnal variation in DIT. The postprandial rise in energy expenditure lasts for several hours and is often regarded as completely terminated at approximately 10 hours after the last meal but there is still an argument as to when the post absorptive state is reached. Reed and Hill [4] analyzed 131 DIT tests from a wide range of subjects ingesting meals of varying sizes and composition. Each test lasted 6 h. They concluded that DIT is a response lasting more than 6 h, especially in obese subjects. Many methodological problems in the measurement of DIT like the choice of meal size and the length of the measurement interval can be circumvented by measuring DIT over 24 h in a respiration chamber [5,6]. Then, activity associated energy expenditure is subtracted from 24 h energy expenditure leaving basal metabolic rate and DIT.

Here, the focus is on DIT as a function of the energy content and nutrient composition of the test food consumed and the duration of the postprandial measurement period in adult subjects with a normal bodyweight. The review is based on literature published over the last 15 years.

## Methods

The experimental design of most studies on DIT is a measurement of resting energy expenditure before and after a test meal, with a ventilated hood system. The observation is started after an overnight fast, where subjects are refrained from eating after the last meal at 20.00 h at the latest. Thus, with observations starting between 08.00 and 09.00 h the next morning, the fasting interval is at least 12 h. Postprandial measurements are made for several hours where subjects have to remain stationery, most often in a supine position, for the duration of the measurements. In some studies, measurements are 30 min with 15 min intervals allowing i.e. for sanitary activities.

The use of a respiration chamber to measure DIT has the advantage of reproducing more physiological conditions over a longer period of time while regular meals are consumed throughout the day [5,6]. The DIT, as observed in a respiration chamber over 24 h has been evaluated in different ways: 1) as the difference in 24-h energy expenditure between a day in the fed state and a day in the fasted state; 2) as the difference in daytime energy expenditure adjusted for the variability of spontaneous activity and basal metabolic rate; and 3) as the difference in 24-h energy expenditure adjusted for the variability of spontaneous activity and basal metabolic rate.

Studies on DIT were selected from Medline. Studies were selected when information was presented on energy intake, diet composition with respect to carbohydrate, protein fat and alcohol of the test food, duration of the postprandial measurement, and DIT.

## Results

Reported intra-individual variability in DIT, determined with ventilated-hood systems, is 6 to 30% [7,8]. Reported within-subject variability in DIT, determined with a respiration chamber, is 43 to 48% [5,9]. The figures for the respiration chamber measurements are for the 24-h DIT calculation as described above under method 3. Method 2, daytime DIT calculation, resulted in an intra-individual variability of 125% [5].

**Figure 1:**
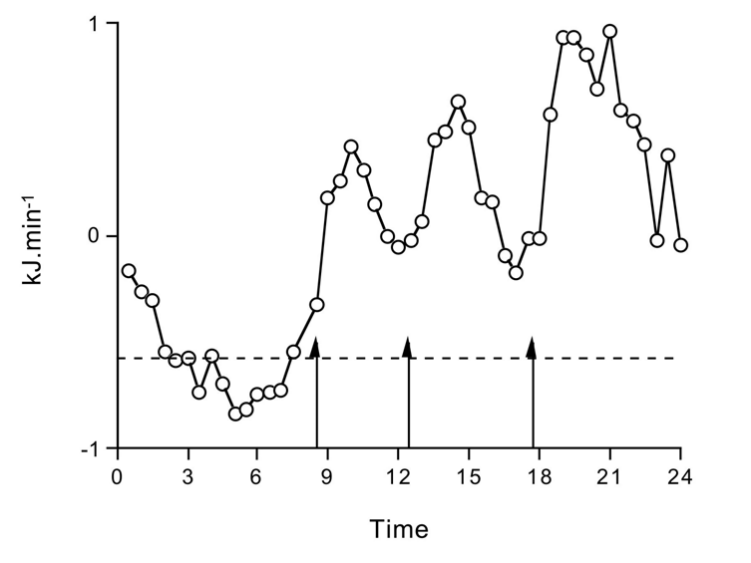
The mean pattern of diet induced thermogenesis throughout the day, calculated by plotting the residual of the individual relationship between energy expenditure and physical activity in time, as measured over 30-min intervals from a 24-h observation in a respiration chamber. Subjects were 17 females and 20 males [10]: -----, level of basal metabolic rate; arrows, meal times.

The mean pattern of DIT throughout the day is presented in figure 1. Data are from a study where DIT was calculated by plotting the residual of the individual relationship between energy expenditure and physical activity in time, as measured over 30-min intervals from a 24-h observation in a respiration chamber [10]. Subjects were 17 females and 20 males. The level of resting metabolic rate after waking up in the morning, and directly before the first meal, was defined as basal metabolic rate. Resting metabolic rate did not return to basal metabolic rate before lunch at 4 h after breakfast, or before dinner at 5 h after lunch. Overnight, basal metabolic rate was reached at 8 h after dinner consumption.

Fifteen studies on DIT with information on energy intake, on diet composition and on the postprandial measurement period were selected from literature (Table 1). Five studies compared DIT, as measured with the same protocol in the same subjects, for two or more diets with a different composition. For alcohol, there was a tendency for an increased DIT, from 7.2 to 8.6 % of the energy content of the meal, when 22% of the energy content of a meal was exchanged for an alcoholic aperitif [11]. In a second study, with a similar energy exchange with alcohol, there was a significant increase in DIT, from 7.1 to 9.0 % of the energy content of the meal [12]. For protein, there was a tendency for and increased DIT, from 7.1 to 8.3% when 20 en% of the meal was exchanged with protein [12]. In a second study, with a similar energy exchange with protein, there was a significant increase in DIT, from 10.5 to 14.6 % of the energy content of the meal [6]. For carbohydrate and fat, one study showed no effect [12], one study showed an increase after the exchange of 65 en% fat for carbohydrate [13], and one study showed the opposite, a decrease after the exchange of 28 en% fat for carbohydrate [14].

**Table 1:** Diet composition, expressed as the energy contribution of carbohydrate, protein, fat and alcohol (en% C/P/F/A) of food intake, and diet-induced thermogenesis (DIT), measured as the increase in energy expenditure above basal fasting level over the presented time interval and expressed as a percentage of the energy content of the food ingested (% intake).

Reference	subjects (n)	diet (en% C/P/F/A)	intake (MJ)	time (h)	DIT (% intake)
[3]	10	57/10/33/0	1.9	4	7.1
[11]	12	45/10/45/0	2.5	4	7.2
	12	35/8/35/22	2.5	4	8.6
[24]	6	0/0/0/100	0.9	4	17.1
[5]	471	50/20/30/0	9.4	24	18
[25]	6	73/11/16/0	2.8	4	4.2
[26]	6	65/10/25/0	2.5	5	6.5
[13]	18	80/18/2/0	2.2	4	4.0^a^
	18	15/18/67/0	2.2	4	5.0^b^
[27]	12	45/15/40/0	3.1	5.5	8.3
[6]	8	30/10/60/0	8.9	24	10.5^a^
	8	60/30/10/0	8.9	24	14.6^b^
[28]	12	45/15/40/0	3.6	5.5	7.1
[29]	24	62/27/11/0	3.8	5	8.1
[14]	12	40/12/48/0	2.5	5	4.3^a^
	12	68/12/20/0	2.5	5	6.5^b^
[30]	14	42/15/43/0	2.5	5	5.8
[31]	13	80/17/3/0	3.2	4	5.2
[12]	19	37/32/31/0	2.8	5	8.3
	19	65/12/24/0	2.8	5	7.1^a^
	19	24/12/65/0	2.8	5	7.1^a^
	19	43/12/24/23	2.8	5	9.0^b^

For a comparison of DIT between studies as a function of the nutrient composition of the test food consumed, the energy content of the test food was divided by the length of the measurement interval after food consumption and expressed in MJ/h. Only three of the 22 studies presented in table 1 included alcohol as a nutrient and were excluded. In a regression analysis of the remaining 19 studies, the protein fraction of the food came out as significant determinant of DIT. An increase in the protein fraction of one % resulted in an increase of DIT with 0.22 ± 0.42 % (p < 0.05).

## Discussion

The main determinant of DIT is the energy content of the food, followed by the protein fraction of the food. The thermic effect of alcohol is similar to the thermic effect of protein.

Diet induced thermogenesis is related to the stimulation of energy-requiring processes during the post-prandial period. The intestinal absorption of nutrients, the initial steps of their metabolism and the storage of the absorbed but not immediately oxidized nutrients [15]. As such, the amount of food ingested quantified as the energy content of the food is a determinant of DIT. The most common way to express DIT is derived from this phenomenon, the difference between energy expenditure after food consumption and basal energy expenditure, divided by the rate of nutrient energy administration [16].

Theoretically, based on the amount of ATP required for the initial steps of metabolism and storage, the DIT is different for each nutrient. Reported DIT values for separate nutrients are 0 to 3% for fat, 5 to 10% for carbohydrate, 20 to 30% for protein [16], and 10 to 30% for alcohol [6]. In healthy subjects with a mixed diet, DIT represents about 10% of the total amount of energy ingested over 24 h. When a subject is in energy balance, where intake equals expenditure, DIT is 10% of daily energy expenditure.

Of the studies presented in table 1, most reported a DIT value below 10% of the energy content of the food ingested. The studies reporting a DIT value below 10% measured DIT as the increase in energy expenditure above basal fasting level over an interval of 4 to 5.5 h after the meal. The studies with a higher value included a study with pure alcohol consumption and the studies where DIT was measured over 24 h in a respiration chamber. In the respiration chamber studies, DIT values were calculated as the increase in energy expenditure above sleeping metabolic rate while the other studies reported DIT as the increase in energy expenditure above basal metabolic rate. Basal metabolic rate is about 5% higher than sleeping metabolic rate [17]. After correction of the DIT values based on sleeping metabolic rate to the increase in energy expenditure above basal metabolic rate, the chamber values are close to the values of 10% of daily energy intake.

The higher DIT value of alcohol and protein compared with carbohydrate and fat has implications for the effect of these nutrients on energy balance. However, the main effect on energy balance does not seem to be primarily linked to the lower bioavailability of alcohol-and protein energy than that of fat and carbohydrate. Alcohol energy is largely additive to the normal diet but does not seem to affect energy balance positively [18]. Protein plays a key role in food intake regulation through satiety related to DIT [19].

Alcohol forms a significant component of many diets and it supplements rather than displaces daily energy intake. Alcohol consumption as an aperitif has even been shown to result in a higher subsequent intake with no intake compensation afterwards [20]. Yet, alcohol intake does not systematically increase body weight. In a recent study, it was shown that subjects with higher alcohol consumption are habitually more active [21]. This may be one explanation for the lack of increasing body weight through additional energy intake from alcohol.

The main effect of protein on energy balance is thought to be DIT related satiety. Satiety scores were higher during meals with a high-protein/high-carbohydrate diet, as well as over 24 h, than with a high-fat diet [22]. The observed DIT related satiety might be ascribed to the high protein rather than the high carbohydrate content of the diet. Postprandial thermogenesis was increased 100% on a high-protein/low-fat diet versus a high-carbohydrate/low-fat diet in healthy subjects [23]. The DIT increases body temperature, which may be translated into satiety feelings. High-protein diets are favored for weight maintenance, also after weight loss, by favoring maintenance or regain of fat-free mass, by reducing the energy efficiency through a higher thermogenesis, and by reducing intake through an increased satiety [19].

In conclusion, the main determinants of diet-induced thermogenesis are the energy content and the protein-and alcohol fraction of the diet. Protein plays a key role in body weight regulation through satiety related to diet-induced thermogenesis.
